# The association between migraine and breast cancer risk: A systematic review and meta-analysis

**DOI:** 10.1371/journal.pone.0263628

**Published:** 2022-02-10

**Authors:** Elahe Hesari, Mozhgan Ahmadinezhad, Maedeh Arshadi, Hosein Azizi, Farzad Khodamoradi

**Affiliations:** 1 Department of Epidemiology and Biostatistics, School of Public Health, Tehran University of Medical Sciences, Tehran, Iran; 2 Research Center of Psychiatry and Behavioral Sciences, Tabriz University of Medical Sciences, Tabriz, Iran; King Abdulaziz University, SAUDI ARABIA

## Abstract

**Background:**

Migraines is likely to play a protective role in the risk of breast cancer. Some studies have shown that there is an inverse relationship between migraine and breast cancer, and some studies have not found an association; therefore, results from previous studies have been inconclusive and we conducted a meta-analysis to evaluate association between migraine and breast cancer.

**Methods:**

PubMed, EMBASE, Scopus and Web of Science were searched to identify studies on the association between migraine and breast cancer from January 1, 2000 through March 12, 2021. The pooled relative risk (RR) and the 95% confidence intervals (CI) was used to measure this relationship by assuming a random effects meta-analytic model.

**Results:**

A total of 10 studies were included. Our study revealed that there was statistically significant inverse relationship between migraine and breast cancer in case-control studies 0.68 [95% CI: 0.56, 0.82], but no significant relationship was found in cohort studies 0.98 [95% CI: 0.91, 1.06]. Also, migraine reduced the risk of ductal carcinoma 0.84 [95% CI: 0.73, 0.96], and lobular carcinoma 0.83 [95% CI: 0.73, 0.96]. With respect to ER_PR status no association between migraine and breast cancer was found. We found no evidence of publication bias.

**Conclusion:**

Our analysis demonstrated a statistically significantly inverse relationship between migraine and total risk of breast cancer only in case-control studies. In summary, cohort studies do not support an inverse association between migraine and incident breast cancer. While in case-control studies, migraine has an inverse association with ductal carcinoma and lobular carcinoma of breast.

## Introduction

The most prevalent malignancy in women is breast cancer [1]. In 2020, there were 2.3 million women diagnosed with breast cancer and 685000 deaths globally [2]. There was shown that higher lifetime exposure to estrogens increase the risk of breast cancer [3]. In numerous studies, postmenopausal women who used estrogen had higher risk of breast cancer [4, 5]. There are two subtypes of breast cancers: estrogen receptor-positive and estrogen receptor-negative [6, 7]. About 70% of breast cancer cases express estrogen receptor alpha (ER) [8, 9].

Migraine is a common primary headache disorder that is more prevalent in women than in men [10, 11] and often begins at puberty and most affects those aged between 35 and 45 years [12]. American Migraine Study published a data that implicated approximately 18% of the female population and 6% the male population suffer from migraine in the United States [10]. Estrogen plays an important role in migraine pathogenesis, but depending on patients past medical history, age, and use of hormonal therapy affect them in different ways. Estrogen causes migraine through two different paths: estrogen withdrawal migraines or migraines associated with fluctuations in estrogen. In general, most of the times estrogen withdrawal, increased the risk of migraine [13]. Due to fluctuating estrogen levels during menarche, menses, pregnancy, and perimenopause, the risk of migraine changes [14, 15]. Migraine can be sensitive to hormones and women with migraine report that attacks associated with their menstrual cycle [16]. Clinical experiments demonstrated that it is decline in plasma estrogen that contributes to the migraine propagation. administration of estrogen during the premenstrual phase was able to prevent menstrual migraine attacks and when estrogen was no longer administered to the women and the plasma levels of estrogen were allowed to drop, the migraine attacks returned [17]. Thus, it is known that female estrogen hormones are involved in the pathophysiology of migraine, but their exact mechanisms of action remain unclear [18].

Estrogen is likely to play a role in the pathogenesis of migraines and breast cancer and studies have shown an association between them. The first case–control study that was conducted in 2008, indicated that patients with a history of migraine had a lower risk of invasive ductal and invasive lobular breast cancer compared to those without migraine [19] and other case–control studies also showed that migraine had an inverse association with the risk of breast cancer [20–22]. However, in some cohort studies, there was no association between migraines and breast cancer [23, 24]. The results of meta-analyzes implicated that there is a statistically significant inverse association between migraine and the risk of breast cancer [24–26]. The interaction between breast cancer and migraine is complex and not fully elucidated [27]. Some studies have shown that there is an inverse relationship between migraine and breast cancer, and some studies have not found an association. An explanation for the inconsistency of results of previous studies can be a small sample size of these studies. Moreover, as regards that new studies conducted in the last five years, our search is more extensive and more databases were searched. Therefore we conducted a meta-analysis to evaluate association between migraine and breast cancer.

## Methods

### Literature search strategy

To identify observational studies on the association between migraine and breast cancer, a comprehensive search was performed of several electronic databases including PubMed, EMBASE, Scopus and Web of Science from January 1, 2000 through March 12, 2021. The search term comprised the following keywords: “breast cancer”, “breast neoplasms”, “breast cancer lymphedema”, “unilateral breast neoplasms”, “inflammatory breast neoplasms”, “mammary neoplasms”, “breast invasive ductal carcinoma”, “metastatic breast cancer”, “migraine”, “migraine disorders”, “basilar type migraine”, “transformed migraine”, “migraine with aura”, “migraine without aura”, “complicated migraine”, “episodic migraine”, “hemiplegic migraine”, “menstrual migraine”, “ophthalmoplegic migraine”, “retinal migraine”, “sporadic hemiplegic migraine”, and “vestibular migraine”. Also, we investigated references of all articles to identify studies were not included during the initial search. The following inclusion criteria were selected for meta-analysis: the study subjects were adult (≥ 18 years old), the study comprised a case-control or cohort study design, the primary outcome was risk of breast cancer, the relative risk (RR) or odds ratio (OR) and the corresponding 95% confidence interval (CI) of the breast cancer associated with migraine were presented and finally, studies published in English. Furthermore, the exclusion criteria were articles include letter to the editor, case report, review and meta-analysis.

### Study selection

**Fig 1:**
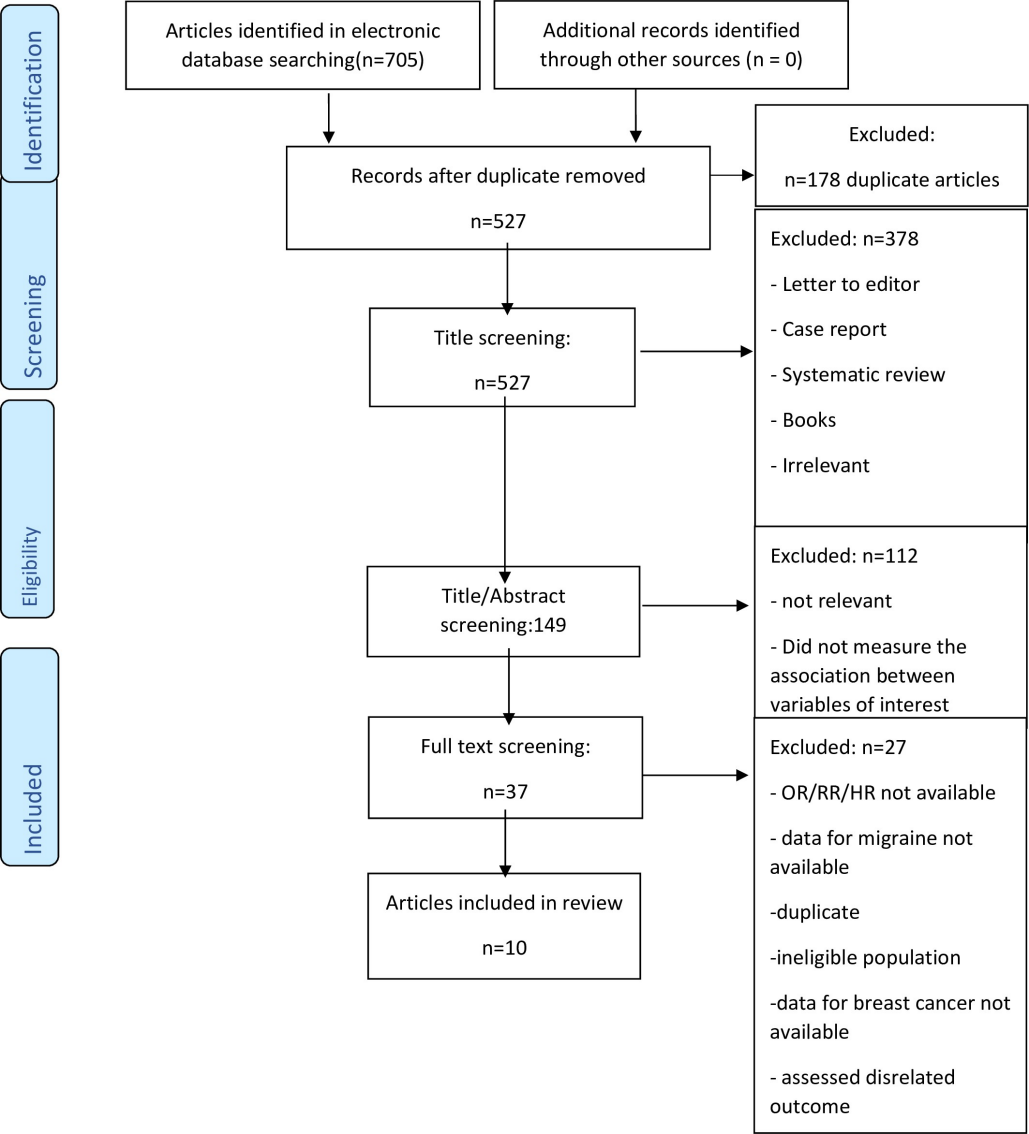
Flow chart depicting the study selection process (screening).

Initially, we screened the titles and abstracts of all studies to identify those that met the inclusion criteria. Full-texts were assessed for studies that were difficult to screen with titles and abstracts only. Two authors (EH and FKH) screened final full texts and decision was made for each study after reading the full texts of all potentially eligible articles. In cases of disagreement, a third review author was consulted or were resolved by discussion. Totally, 705 articles were retrieved, of which a total of 10 articles remained after the review process shown in Fig 1.

### Data extraction

A structured data extraction form was used to extract data from the papers. The extracted data included: last name of the first author, publication year, country, study design, study purpose, sample characteristics, sample size, mean age, confounders. Extraction of data was done by same two review authors (EH and FKH) who conducted the study selection independently.

### Evaluating the quality of articles

The quality of studies was assessed using Newcastle—Ottawa quality assessment scale (NOS) adapted for observational studies [28]. The NOS consist of three domains. These domains include selection of study groups, comparability of groups and description of exposure and outcome. This scale including eight items and star scores assesses the quality of each study in each domain. Except comparability domain other items have one star (maximum score based on stars for comparability domain is two). Totally, for each study earned stars calculated as total quality score. Based on these criteria, study quality was rated on a scale from one star, very poor, to 10 stars, high quality. Studies are rated as of high (7–10), medium (5–6) or low quality (< 4). Two review authors (EH and FKH) completed quality assessment independently. A third review author was involved in cases of disagreement.

### Statistical analysis

The pooled RR and the 95% confidence intervals were used to measure the association between migraine and the risk of breast cancer by assuming a random effects meta-analytic model. We used adjusted estimates and assumed that the odds ratios (ORs) from case-control studies approximated hazard ratios. Statistical heterogeneity was evaluated using Cochran’s Q-test and I^2^ statistic_._ Subgroup analysis carried out according to the study design (case-control or cohort), hormone receptor status (ER+_PR+ or ER+_PR- or ER-_PR-), and histologic subtype (lobular vs ductal). Leave-one-out sensitivity analysis was performed to identify influential studies in meta-analysis. Publication bias was determined by funnel plot and Begg’s and Egger’s tests. The p value of <0.05 considered as statistically significant. The analyses were performed using Stata software version 14.

## Results

### Study characteristics

Search strategy and the algorithm of study selection are shown in Fig 1. According to the keywords, MeSH terms and Emtree terms a total of 705 studies were identified. Subsequently, after identifying relevant studies and removing duplicates and considering the inclusion and exclusion criteria, 378, 112, and 27 studies were excluded after reviewing their titles, abstracts, and full-texts, respectively. Finally, 10 related studies in quality analysis were evaluated and received inclusion criteria. Of these, 5 were cohort and 5 were case-control studies. Seven studies were conducted in the United States, one study in Taiwan, one study in Norway and one study in Iran. The cut off score of 7 or higher was considered as the studies with high quality and 5–6 was considered as the studies with moderate quality. Five studies were in range of 7–10, that they had high levels of quality. Five studies were in range of 5–6, that had moderate levels of quality. Table 1 summarizes characteristics of selected studies.

**Table 1:** Characteristics of included studies in the meta-analysis.

Author/year	country	Study design	Study purpose	Sample characteristics	Sample size	Mean age/range	Main measurements	confounder	NOS
Fan et al., 2018 [23]	Taiwan	cohort	investigate the association between migraine and breast cancer incidence	Woman with newly diagnose migraine and woman without migraine from the National Health Insurance Research Database in Taiwan	25,606 with migraine,	43.4	HR = 1.03, 95% CI = 0.89, 1.21	age, urbanization level, comorbidities, medications, numbers of breast cancer screening examinations	7
					102,424 without migraine				
C. Winter et al., 2015 [24]	US	Cohort	evaluated the association between migraine and breast cancer risk	From the Nurses’ Health Study II (NHS II)	97 682 with Migraine,	25–42	HR = 0.96, 95% CI = 0.88, 1.04	age, BMI,	5
								History of breast cancer, parity, Age at first birth, breast feeding use, age at menarche, smoking status, alcohol, menopausal status, estrogen only use, estrogen and progesterone use, other hormone use	
					17 696 without migraine				
Min Shi et al., 2015 [37]	Norway	Cohort	examine the possible associations between breast cancer and migraine overall	The Sister Study recruited women in the United States and Puerto Rico and had a sister with breast cancer	10766 with migraine,	35–74	HR = 0.98, 95% CI = 0.89,1.07	race, age at menarche, BMI, age at first birth and menopause status	7
					41507 without migraine				
C. Winter et al., 2013 [30]	US	cohort	evaluate the association between migraine and incident breast cancer	Women’s Health Study	7,318 with migraine,	45<	HR = 1.10, 95% CI = 0.99,1.22	age, BMI, alcohol consumption, smoking status, postmenopausal status, age at menarche, age at menopause, postmenopausal hormone use, number of pregnancies, age at first pregnancy, family history of breast cancer, history of benign breast disease	5
					32,378 without migraine				
Li et al., 2010 [32]	US	cohort	assessed the relationship between self-reported history of migraine and incidence of postmenopausal breast cancer	Women’s Health Initiative Observational Study prospective cohort	10,464 with migraine, 80,652 without migraine	50–79	HR = 0.89, 95% CI = 0.88, 98	age, race/ethnicity, hysterectomy, use of menopausal hormones, nonsteroidal anti-inflammatory drug use/duration, alcohol consumption, smoking status, and regular coffee consumption	5
Mathes et al., 2008 [19]	US	case-control	examine the relationship between migraine and risk of postmenopausal invasive breast cancer	women diagnosed with invasive breast cancer between 1997 and 1999 regardless of histologic type.	1938 cases 1,474 controls	55–79	OR = 0.67, 95% CI = 0.57, 0.80	Age, reference year	7
Li et al., 2009 [21]	US	Case control	to evaluate the relationship between a history of migraine and risk of breast cancer	The Women’s Contraceptive and Reproductive Experiences Study is a population-based case-control study that recruited women diagnosed with invasive breast cancer between 1994 to 1998 from five metropolitan areas: Atlanta, Detroit, Los Angeles, Philadelphia, and Seattle	4,568 cases 4,678 controls	38–64	OR = 0.74, 95% CI = 066, 0.82	age, race, and study site	7
Lowry et al., 2014 [22]	US	Case- control	Identifying specific characteristics of migraines which are most strongly associated with breast cancer risk	Cases were women diagnosed with a primary invasive ductal or lobular breast cancer and Population-based controls were then identified by random-digit dialing within the same three counties of residence as cases	715 cases	55–74	OR = 1.00, 95% CI = 0.70, 1.50	age, county of residence, reference year, body mass index	8
					376 controls				
Whiteman et al., 2010 [40]	US	case-control	migraine history is associated with a reduced risk of breast cancer	Cases were women newly diagnosed with breast cancer. Women in the same age range were selected as controls using random-digit dialing.	4701 cases	20–54	OR = 0.77, 95% CI = 0.68, 86	smoking, alcohol use, exogenous hormone use	5
					4666 controls				
Ghorbani et al., 2015 [20]	Iran	Case- control	assess and compare the relative frequency of migraine between breast cancer sufferers	The case group consisted of 400 women, with a positive history of breast cancer, registered at the oncology center of Isfahan University of Medical Sciences. Women of the control group were enrolled through cluster sampling among women registered in five cultural centers of different areas of Isfahan.	400 cases	20–60	OR = 0.37, 95% CI = 0.27, 49	NA	6
					400 controls				

CI = confidence interval; HR = hazard ratio; NOS = Newcastle—Ottawa quality assessment scale; OR = Odds ratio; NA = Not available.

### Types of migraine

In Ghorbani et al. study, the diagnosis of different types of headaches was made based on The International Headache Society (IHS) guidelines. Min Shi et al. examine the possible associations between breast cancer and migraine overall and between cancer subcategories and the two migraine subtypes; menstrually-related from non-menstrually-related migraine. C winter et al. and Lowry et al. considered migraine characteristics (i.e., migraine subtypes, migraine with aura or migraine without aura). Migraine with aura was defined as at least two headaches accompanied by any visual disturbances such as flickering lights, spots, and lines or loss of vision before or during a headache, not attributed to other disorders [30]. In other included studies, once a woman reported a diagnosis of migraine, she was considered a migraineur.

### Migraine and breast cancer risk

**Fig 2:**
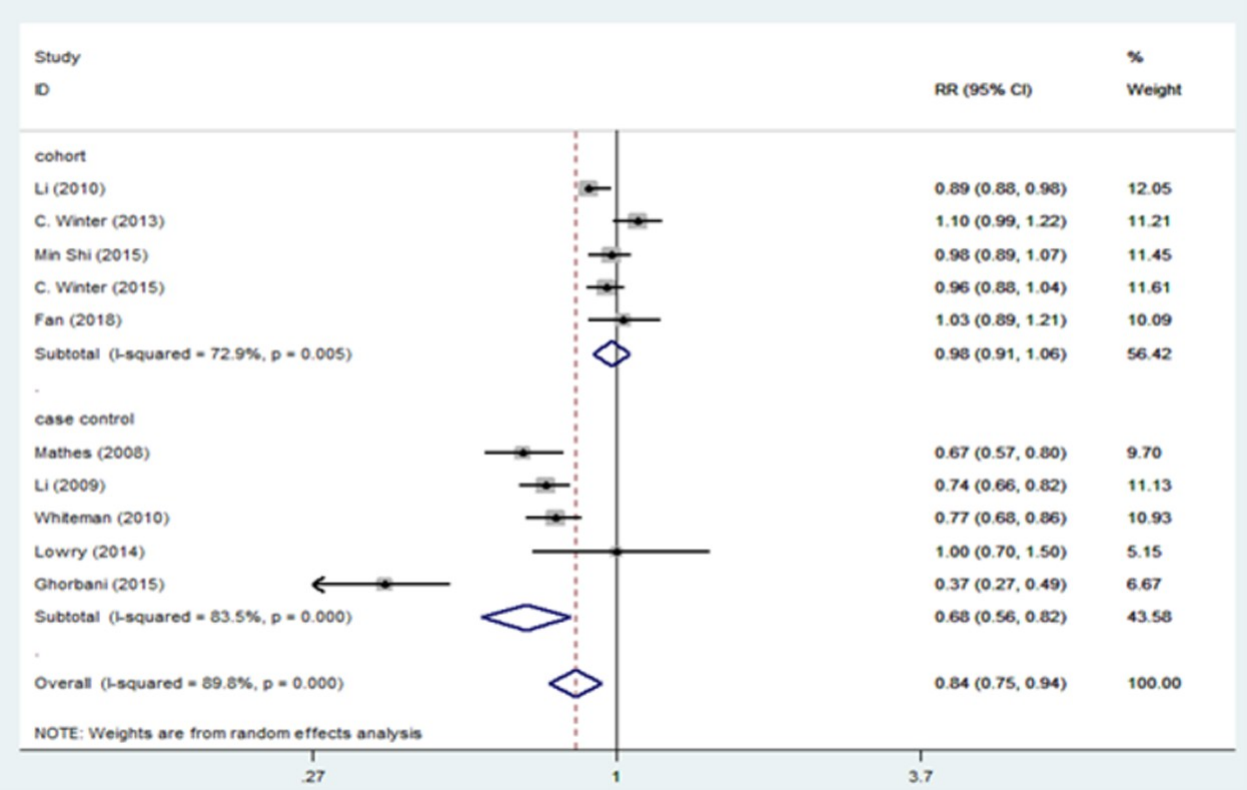
Forest plot of the association between migraine and breast cancer by study design.

**Fig 3:**
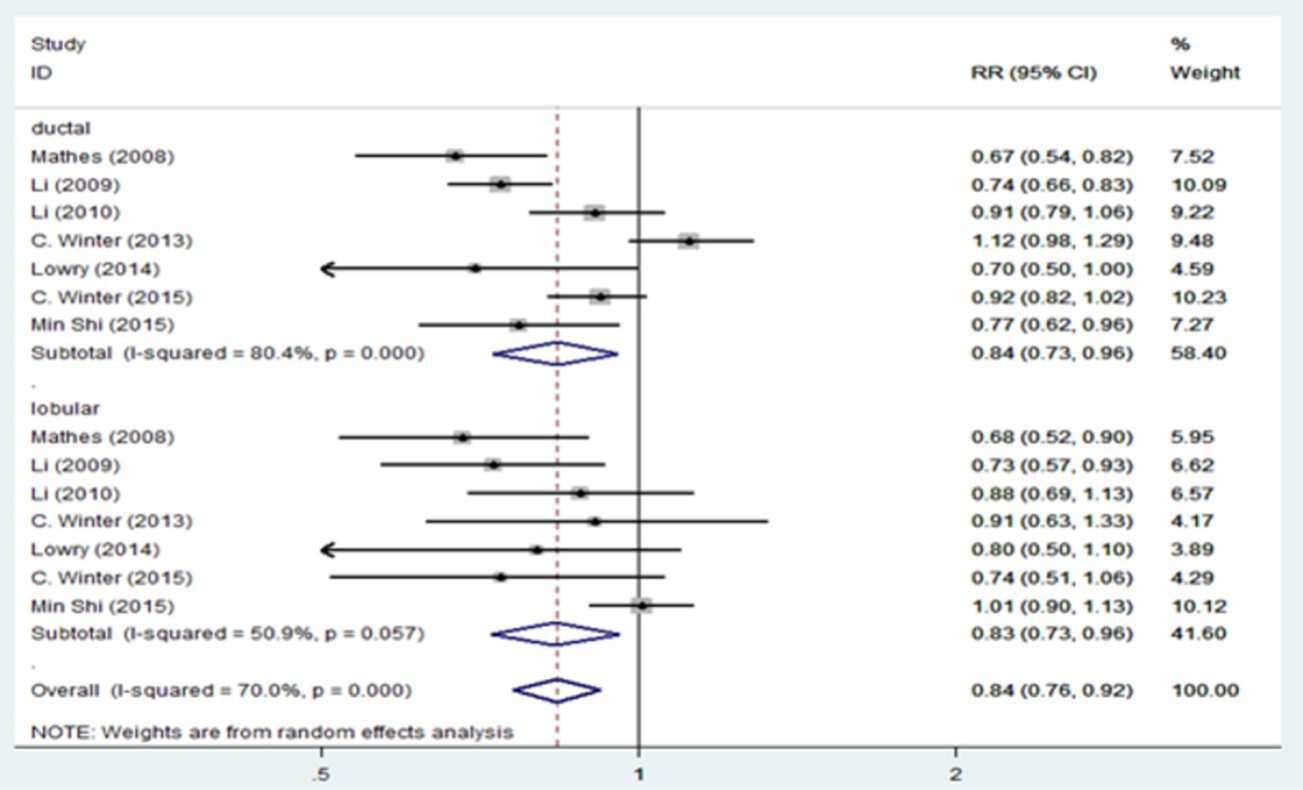
Forest plot of the association between migraine and breast cancer by histologic subtype.

**Fig 4:**
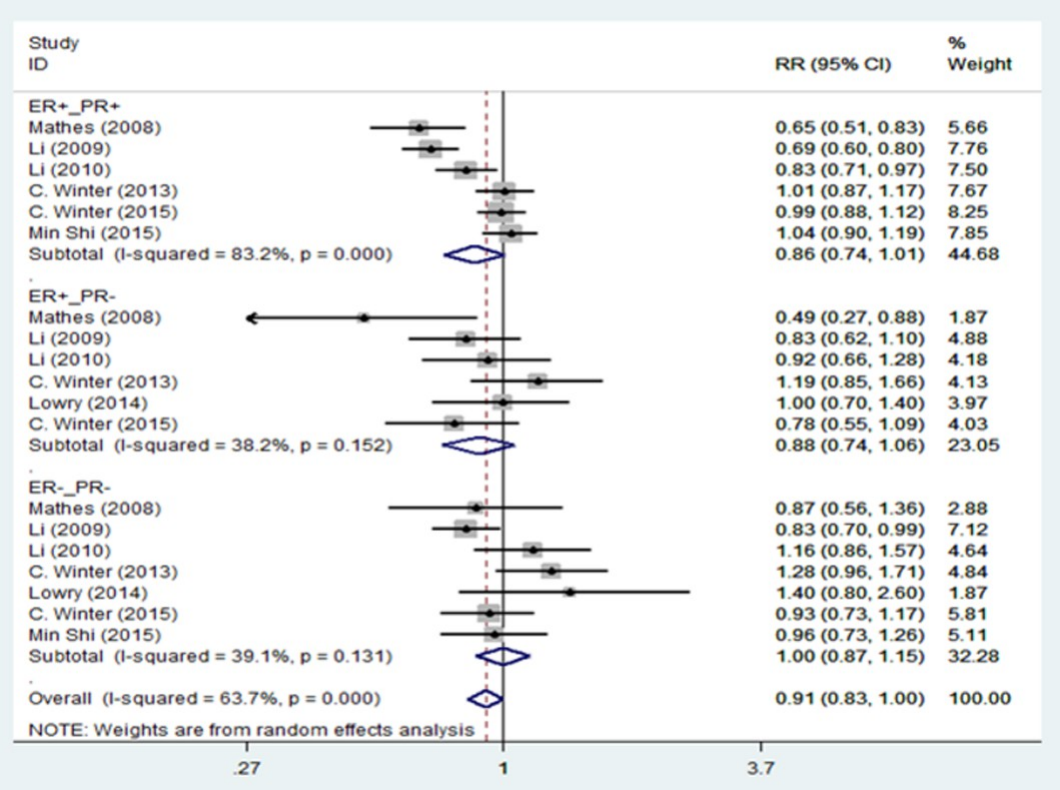
Forest plot of the association between migraine and breast cancer by hormone receptor status. CI = confidence interval; ER = estrogen receptor; PR = progesterone receptor; RR = relative risk.

Fig 2 presents the results of the random-effects meta-analysis and the pooled adjusted RR for association of migraine and breast cancer stratified by study design. Based on results, there is a statistically significant inverse relationship between migraine and breast cancer in case-control studies 0.68 [95% CI: 0.56, 0.82], but no significant relationship was found in cohort studies 0.98 [95% CI: 0.91, 1.06]. We revealed a statistically significant inverse relationship between migraine and total breast cancer risk 0.84 [95% CI: 0.75, 0.94]. However, there is evidence of significant heterogeneity among cohort studies (I^2^ = 72.9%; P = 0.005) and case-control studies (I^2^ = 83.5%; P = 0.000) and among all studies (I^2^ = 89.8%; P = 0.000). Fig 3 presents the pooled adjusted RR for association of migraine and breast cancer stratified for ductal and lobular breast cancer. Based on this figure, migraine reduced the risk of ductal carcinoma 0.84 [95% CI: 0.73, 0.96], and lobular carcinoma 0.83 [95% CI: 0.73, 0.96]. There is significant heterogeneity among ductal breast cancer studies (I^2^ = 80.4%; P = 0.000), but heterogeneity decreased among lobular breast cancer studies (I^2^ = 50.9%; P = 0.057). The results of the relationship between migraine and breast cancer stratified by hormone receptor status are shown in Fig 4. According to ER and PR status, no association was found between migraine and breast cancer. There is no evidence of heterogeneity in the ER+_PR- (I^2^ = 38.2%; P = 0.152) and ER-_PR- (I^2^ = 39.1%; P = 0.131) groups.

**Fig 5:**
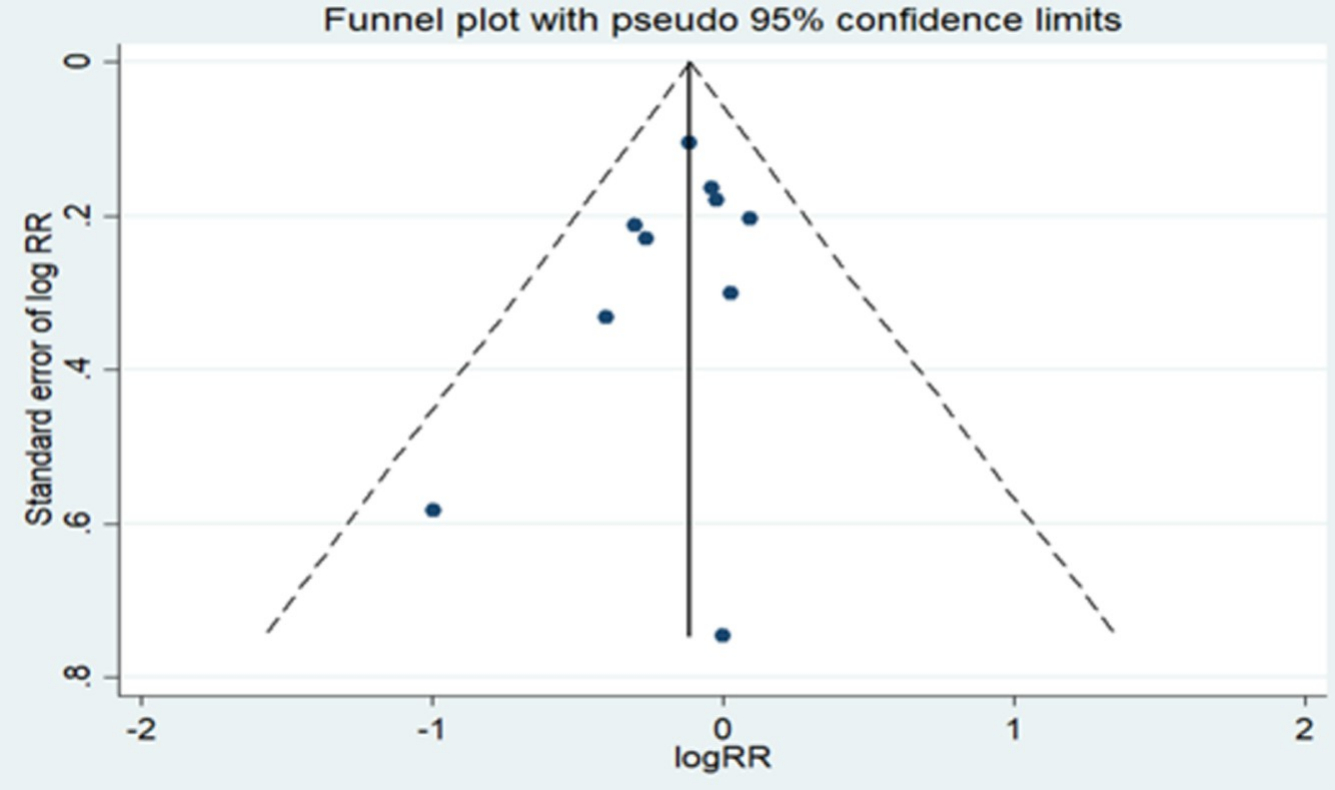
Funnel plot for publication bias.

In addition, Leave-one-out sensitivity analysis was performed to identify influential studies on pooled RR in meta-analysis. Our results showed that no single study significantly changed the pooled RR. Overall, we determined the possibility of publication bias using the funnel plot (Fig 5) as well as Begg’s and Egger’s tests. The studies almost symmetrical scattered on both sides of the vertical line showing the absence of publication bias. Based on the Begg and Egger tests (Pb = 0.325 and Pe = 0.283), we found no evidence of publication bias.

## Discussion

The relationship between migraine and breast cancer was first studied in 2008 in a study by Mathes et al. the study reported that a history of migraine is associated with a decreased risk of breast cancer, particularly among ER+ /PR+ ductal and lobular carcinomas [19]. A study published in the Journal of Clinical Oncology in 2010 also found that women with a history of migraine had a lower risk of breast cancer (HR = 0.89) than women without a migraine history. The lower risk was somewhat more pronounced for invasive estrogen-receptor–positive and progesterone-receptor–positive tumors as no reduction in risk was observed for invasive ER-negative/PR-negative tumors [29]. Later, more detailed studies were conducted to investigate this relationship. A cohort study in 2014 examined more than 700 cases of breast cancer. During a mean-follow-up time of 13.6 years, migraine was not associated with breast cancer risk. The multivariable-adjusted HRs were 1.10 for any breast cancer [30]. In meta-analysis that conducted among 115378 Nurses’ Health Study in 2015, in cohort studies, migraine was not associated with breast cancer risk or differences in endogenous sex hormone levels. While case-control studies suggest an inverse association between migraine and breast cancer risk [24]. Furthermore, Fan et al. indicated that women with ≥4 medical visits for migraine per year had a significantly greater risk of breast cancer than the matched cohort [23]. These articles suggest the relationship between breast cancer and migraine in women’s health, but there are conflicting results between studies that highlight the need for a new meta-analysis study that includes recent studies to have a summary of the conclusions.

The relationship between migraine and breast cancer is still unclear because both migraine and breast cancer are associated with estrogen and the mechanism of action of estrogen is very complex [5]. Also one of the reason for these conflicting results is that studies have been conducted in different communities that they may have different factors that can affect breast cancer; For example, among women with migraine, independent risk factors for breast cancer included older age, alcohol-related illness, and receipt of a greater number of breast cancer screening examinations, and independent protective factors included the use of antihypertensive agents, statins, and nonsteroidal anti-inflammatory drugs [23].

In the present study, 10 articles were identified and included from PubMed, ISI, Embase and Scopus for a meta-analysis that concerns the association between migraine and the risk of breast cancer. Our analysis demonstrated a statistically significantly inverse relationship between migraine and the total risk of breast cancer. The evidence indicative of strong heterogeneity was positively detected among studies which could be due to differences in ages, study design, adjustment for confounding factors, and other unknown factors.

Although a statistically significantly inverse relationship has been observed between breast cancer and migraine, its biological mechanisms is unclear [31]. Migraine patients use variety of analgesics including nonsteroidal anti-inflammatory drugs (NSAIDs). Many studies indicate that NSAID use may result in diminished risk of breast cancer [32–34]. It seems that people with headache have lesser risk of cancer because of long-term use of NSAIDs. Furthermore, maybe this happen to breast cancer sufferers too, by taking antidepressant medications or analgesics as a part of breast cancer management. However, it needs more studies to identify the exact cause [20].

Another explanation for a lower risk of breast cancer in women with a history of migraines could be that such women are more likely to avoid migraine triggers (i.e., alcohol, cigarette smoking, poor sleep, stress), some of which are also risk factors for breast cancer [19, 21, 32]. Such behaviors might be expected to lower the risk of ER+ breast cancer. Also, migraines are more frequent in women than in men [35, 36], and among women they are more common during the years of menstruation within 2 days of the start of the menstrual cycle, and in oral contraceptive users [10].

Subgroups analysis by study design (cohort and case control), Histological subtype (ductal and lobular breast cancer) and hormone receptor (ER+/PR+, ER+/PR− and ER−/PR−) was performed. Notably, such an inverse relationship was identified in the case–control studies [19, 20, 22], but no significant relationship was found in cohort [23, 30, 32, 37]. For histological subtype, migraine reduced the risk of ductal carcinoma and lobular carcinoma. Also, estimates demonstrated that migraine was more protective for lobular than for ductal carcinomas. One of these reasons could be that the most common type of breast cancer begins in the breast ducts (invasive ductal carcinoma). Therefore, the number of lobular patients is more. With respect to ER_PR status no association between migraine and breast cancer was observed [38, 39].

The results of our study were similar to previous meta-analysis studies that found a statistically significantly inverse association between migraine and the total risk of breast cancer [26]. Also, in this study, as in previous meta-analyzes, a statistically inverse association between migraine and breast cancer was seen in case control studies [25]. This relationship was not seen in cohort studies [24]. In addition, an inverse relationship between migraine and histological subtype (ductal and lobular breast cancer) was seen [24–26]. Although previous studies have seen this relationship between migraine and breast cancer with respect to ER+/PR+ status [24–26], but in our findings, there was no association between migraine and risk of breast cancer with respect to ER_PR status. One possible reason could be that we included recently published studies in our study. The recent studies that have entered in our study are cohorts and have a larger sample size than the previous studies, which may be one of the reasons for the statistical insignificance.

We used the Q-test and I^2^ statistic to detect of heterogeneity. The evidence indicated that strong heterogeneity was detected among studies, but in lobular breast cancer significant decrease in heterogeneity was observed and no evidence of heterogeneity in the ER+_PR- and ER-_PR- groups was found. There can be various reasons for heterogeneity between studies. The first reason could be the attributed to the number of studies (including ten studies) in the meta-analysis. The second reason could be the difference in sample size of different studies. The third reason for heterogeneity could be publication year. The eligible studies were published from 2000 to 2021. The fourth reason could be related to the geographical area of the published studies. Most of the studies were in the United States. Also, differences in instrumental, methodology (because our meta-analysis include cohort and control case studies) and study population may be other sources of heterogeneity. Finally, the studies that we examined are prone to measurement bias and selection bias.

Our study had some limitations. First, we included studies written in English language. Second, due to the limited number of studies, we have combined cohort and case control studies. Third limitation of this study could be that in case–control studies, information may not have been accurate because it was obtained through self-reporting and there is a possibility of recall bias. Fourth, the studies that reviewed were from 2000 to 2021 and we did not include studies before 2000. Finally, some variables, such as age, that may affect heterogeneity due to limited information have not been studied.

## Conclusion

Our analysis demonstrated a statistically significantly inverse relationship between migraine and the total risk of breast cancer. In summary, results from this meta-analysis demonstrated that cohort studies do not support an inverse association between migraine and incident breast cancer. While case-control studies suggest an inverse association between migraine and breast cancer risk. The results of meta-analysis studies are valuable. Therefore, researchers can use this study for future research.

## Supporting information

S1 Checklist PRISMA 2020 checklist. (DOCX)
